# The influence of calcium and magnesium on body weight: an experimental approach with growing Wistar rats

**DOI:** 10.1186/1758-5996-7-S1-A150

**Published:** 2015-11-11

**Authors:** Amariles Diniz Ramires, Pedro Cassino de Carvalho, Silvio Assis de Oliveira, Jose Antonio Braga Neto

**Affiliations:** 1 Universidade Federal de Mato Grosso do Sul, Barra do Garças, Brazil

## Background

The increase in body weight is a serious global public health problem, with high risk of associated diseases such as diabetes, cardiovascular disease, hypertension, and some forms of cancer. The option for foods with high energy value but poor in micronutrients increases the nutritional status and contributes to the deficiency of important minerals in human health. The use of minerals, among them calcium and magnesium, to control body weight has been the object of several studies.

## Objective

The purpose of this study was to investigate the effect of with the calcium and magnesium supplementation in diets offered to Wistar rats.

## Materials and methods

A biological assay was conducted with 24 animals, distributed in four groups of six. The control group (GI) received feed prepared according to the AIN-93G standard (19.74% protein, 7.48% lipid, 52.64% carbohydrate, and 3.5% saline mixture, with 5000 mg of calcium and 500 mg of magnesium per kilogram of feed); the calcium-supplemented group (GII) received this diet containing approximately four times this mineral; the group supplemented with magnesium (GIII) received approximately four times this mineral; and the lipid-supplemented group (GIV) received it with a 14% addition of vegetable oil, but no mineral supplementation. The diets were isocaloric (3.57 kcal/g). The variables measured were weight, feed efficiency coefficient, liver and kidney histology, and the following laboratory parameters: total cholesterol (TC), high-density lipoprotein (HDL), low-density lipoprotein (LDL), triglycerides (TG), and serum calcium and magnesium levels.

## Results

After 35 days, the GII animals showed the lowest level of feed efficiency, when compared with the other groups, and consequently exhibited lower body weight, with a significant increase in VLDL, TG and serum calcium levels and a decrease in serum magnesium. The morphological analysis of liver and kidney revealed tissue damage in all groups that received supplementation.

## Conclusion

The supplementation with calcium proved a possible resource for body weight control, in contrast with that observed in the animals that received magnesium supplementation, whose results were similar to those of controls.

